# Prevalence of primary headache disorders in a population aged 60 years and older in a rural area of Northern China

**DOI:** 10.1186/s10194-016-0672-3

**Published:** 2016-09-13

**Authors:** Yajing Zhang, Zhihong Shi, Duncan Hock, Wei Yue, Shuling Liu, Ying Zhang, Shuai Liu, Lei Zhao, Hui Lu, Yalin Guan, Xiaodan Wang, Thomas Wsiniewski, Yong Ji

**Affiliations:** 1 Department of Neurology, Tianjin Huanhu Hospital, Tianjin, China; 2 Tianjin Key Laboratory of Cerebrovascular and Neurodegenerative Diseases, Tianjin Huanhu Hospital, Jizhao Road 6, Tianjin, Jinnan 300060 China; 3 Morsani College of Medicine, University of South Floride, Tampa, USA; 4 Departments of Neurology(Aging and Dementia Division), Pathology and Psychiatry, NYU Pearl Barlow Center of Memory Evaluation and Treatment, New York, NY USA

**Keywords:** Primary headache, Prevalence, Tension-type headache, Migraines, Chronic headache, Unclassified headache

## Abstract

**Background:**

Primary headache disorders are among the most common neurological complaints worldwide, and are significant causes of disability. Data are limited on the prevalence of primary headaches among individuals aged 60 years and older in China. The purpose of our study was to investigate the prevalence and characteristics of primary headaches among people aged 60 years and older in a rural area of northern China.

**Methods:**

A door-to-door survey was conducted in Ji County of Tianjin Province. A total of 5248 residents aged 60 years and older were visited from 2014 to 2015, unannounced, and basic sociodemographic and headache diagnostic information was collected via a questionnaire. Primary headache was classified tension-type headaches, migraines, chronic headache, and unclassified headache.

**Results:**

The 1-year prevalence for primary headaches in Ji county is 10.30 %. The 1-year prevalence of tension-type headaches, migraines, chronic headache, and unclassified headache is 2.02 %, 0.85 %, 3.79 %, and 3.63 %. The 1-year prevalence of primary headache was higher in women than in men. The 1-year prevalence of primary headache was decreased with increasing age. The 1-year prevalence of tension-type headaches and chronic headache were decreased with higher education. Headache in the elderly was mild to moderate and not serious.

**Conclusions:**

In a population older than 60 years of age in rural, northern China, we found the 1-year prevalence of primary headache was 10.30 %, of tension-type headaches was 2.02 %, of migraines was 0.85 %, of chronic headache was 3.79 %, and of unclassified headache was 3.63 %. The prevalence of all of these was much lower than those observed in previous studies examining prevalence rates in the general population. The results of our study also indicated that women are more likely to have primary headache than men. We also found that chronic headache was frequent among the elderly. Participants with higher education levels were more predisposed than healthy participants to tension-type headaches. We also found that respondents who did not participate in social activities had a decreased predisposition for primary headache in general, as well as for unclassified headache. Lastly, we found that participants who had heart disease were more likely than healthy participants to experience primary headache, tension-type headache, chronic headache, and unclassified headache.

## Background

Primary headache disorders are among the most common neurological complaints worldwide, and are significant causes of disability. Among all primary headache disorders, tension-type headaches (TTH) are the most common, followed by migraines (MIG). Studies suggest that global 1-year prevalence rates are 46 % for all primary headache disorders, 42 % for TTH, 11 % for migraines, and 3 % for chronic daily headaches (CDH) [1, 2]. The global lifetime prevalence for all primary headaches is 64 % [3].

Peng et al. reported the 1-year prevalence of primary headache disorders in the Asia-Pacific regions 9.1 % for MIG, 16.2 % for TTH, and 2.9 % for CDH [4]. Comparatively, Stovner et al. found the 1-year prevalence of primary headaches in Europe to be 51 %, while MIG was 14 %, and CDH was 4 % [5]. In a door-to-door population-based survey in China, the 1-year prevalence of primary headache disorders was 23.8 %, and was higher among females. The 1-year prevalence of migraine was 9.3 %, while TTH was 10.8 % [6]. Another survey showed that headache disorders are common, burdensome, and very costly in China [7].

The Chinese population is aging; 15.5 % of China’s citizens were aged 65 years or older in 2014. It is estimated that the percentage will reach 18.2 % by 2030, and the elderly will constitute an even larger segment of this society. Multiple epidemiological studies show headache prevalence decreases in elderly population [5, 8, 9]. Headache etiology and presentation are considerably different in elderly individuals. However, data are limited on the prevalence of primary headaches among individuals aged 60 years and older in China.

The purpose of our study was to investigate the prevalence and characteristics of primary headaches among people aged 60 years and older in a rural area of northern China. The diagnostic criteria were based on the International Classification of Headache Disorders (ICHD), third edition.

## Methods

### Data source

A door-to-door survey was conducted in Ji County of Tianjin Province. A total of 5248 residents aged 60 years and older were visited from 2014 to 2015, unannounced, and basic sociodemographic and headache diagnostic information was collected via a questionnaire. The questionnaires were administered by qualified and experienced specialists in headache neurology, all of who were trained at Tianjin Huanhu Hospital in Tianjin, China, using the ICHD-3-beta criteria and the questionnaire.

All elderly (≥60 years) residents (approximately 5,248 individuals) in 26 villages of Ji County were visited. A population sample of residents in Ji County of Tianjin Province older than 60 years was selected and subsequently evaluated using an 18-item questionnaire. Among the 5248 residents interviewed, 106 were not at home and thus could not fill out the questionnaire, 54 declined to complete the questionnaire, and 50 submitted an incomplete survey. These included people unable to provide reliable information about their headache and residents who had cognitive decline of normal aging or the more serious decline of dementia. A total of 5038 residents completed the questionnaire, and the response rate was 96 %. The age of the respondents ranged from 60 to 96 years, and the average age was 71.2 ± 17.5 (mean ± SD) years.

Prior to conducting this survey, a pilot study surveyed one village to test the epidemiological methods.

The questionnaire was designed to classify headache types according to the criteria of the International Classification of Headache Disorders, third edition [10]. The questionnaire included questions to determine a headache profile as well as basic demographic and socioeconomic information. The headache profile included the following: headache nature (pulsating, aching, feeling distending pain/tightening, etc.), duration (<30 min, 30 min to 4 h, 4–72 h, ≥72 h, and undetermined), associated symptoms (nausea, vomiting, or both; phonophobia, photophobia, or both; lacrimation or conjunctival injection; not reported), attack frequency (occasional,<1 day/month; chronic, ≥15 days/month; episodic, >1 day/month and <15 days/month; everyday), severity (based on a visual analog scale [VAS] score of 0 to 10, in which 1–3 represented mild pain, 4–6 moderate pain, and ≥7 severe pain), the impact of physical activity on headache, and family history.

The questions regarding demographic and socioeconomic characteristics were basic and included age, sex, education level (illiterate, schooling ≤5 years, and schooling >5 years), marital status (married, widowed, or separated), social activities (no social activities versus socially active), and comorbidities (hypertension, diabetes mellitus, and heart disease).

The headache profile and demographic sections of the questionnaire consisted of the same items as those used in a Chinese national epidemiology study, and were validated for headache diagnosis and assessment in the general population [6, 11]. Secondary headaches, which resulted from trauma, vascular disorders, infection, cranial neuralgias, facial pain, and psychiatric disorders, were excluded. We used the ICDH-3-beta to exclude secondary headache. People who reported headache were further examined to determine the presence of primary or secondary headache. This assessment was performed during a second home interview, which was conducted by two of six board-certificated neurologists from Tianjin Huanhu Hospital. The six neurologists had been given training together to ensure consistent neurological consultation of all participants. A detailed medical history was obtained from all patients, and each patient received a physical and neurological examination. If there was still uncertainty, a brain MRI was conducted to exclude intracranial lesions.

Primary headache was classified into TTH, MIG, chronic headache (CH), and unclassified headache (UNC). TTH and MIG were diagnosed based on criteria described in the ICDH-3-beta. UNC included cluster headache (the symptoms of only two respondents conformed to this diagnosis), trigeminal autonomic cephalalgia, medication overuse headache (because the number of this subtype was very small, it was not classified as a separate group) and headache that could not be classified because the manifestations were not typical.

MIG criteria included an attack lasting 4–72 h and accompanied by any two of the following manifestations: unilateral location, pulsating quality, moderate-to-severe intensity, and aggravated by routine physical activity. The attacks also must have been accompanied by at least one of the following: nausea or vomiting, or both photophobia and phonophobia. Additionally, at least five attacks must have occurred in a lifetime.

The diagnosis of TTH required more than 10 attacks in a lifetime. Each attack had to last 30 min to 7 days and had to be associated with at least two of the following manifestations: bilateral location, non-pulsating quality, mild-to-moderate intensity, and non-aggravation by routine physical activity. TTH was not accompanied by nausea or vomiting, but could include either photophobia or photophobia, but not both.

The diagnosis of CH (chronic headache) included attacks occurring at a frequency of more than 15 days/month over 3 months, with the attack lasting more than 4 h per day [12, 13].

We selected the “most troublesome headache” as the main headache when a respondent fulfilled criteria for more than one type of headache. After the survey, a subsample of respondents was randomly selected from all the respondents and asked to undergo second interviews. Each of these second interviews was conducted via telephone by a neurologist, who used his or her neurological expertise to determine an appropriate diagnosis.

### Statistical analysis

The diagnosis of primary headache was based on ICHD-3 criteria. Based on this diagnosis, the 1-year prevalence, using a 95 % confidence interval (CI), is presented as the number of cases per 100 persons. Age- and sex-specific prevalence rates were also calculated. The results were analyzed using statistical software SPSS 16.0. Student’s *t*-test, chi-square test, and analysis of variance were used for comparisons when appropriate. Multivariate logistic regression was used to identify odds ratios (ORs) and 95 % CIs for headache subtypes. Missing data resulting from nonresponse occurred for several variables. The reported data are based on the available data. The statistical significance was set at *P* < 0.05.

## Results

Table 1 summarizes the results of the 1-year prevalence of primary headache according to sex, age, and education. Among the 5038 residents (60 years of age and older) who we interviewed, The 1-year prevalence for primary headaches in Ji county is 10.30 % (95 % CI 9.46–11.14). The 1-year prevalence of TTH, MIG, CH, and UNC is 2.02 % (95 % CI 1.64–2.41), 0.85 % (95 % CI 0.60–1.11), 3.79 (95 % CI 3.26–4.32) and 3.63 (95 % CI 3.12–4.15).

**Table 1:** Sex-, age- and education-specific 1-year prevalence of headache types (%)

	N^a^	All primary headache			TTH			MIG			UNC			CH		
		No cases	Prev^b^(%)	95 % CI^c^	No cases	Prev (%)	95 % CI	No cases	Prev^b^(%)	95 % CI	No cases	Prev (%)	95 % CI	No cases	Prev (%)	95 % CI
Total	5038	519	10.30	9.46–11.14	102	2.02	1.64–2.41	43	0.85	0.60–1.11	183	3.63	3.12–4.15	191	3.79	3.26–4.32
Gender																
Male	2366	125	5.28	4.38–6.18	33	1.39	0.92–1.87	5	0.21	0.03–0.40	41	1.73	1.21–2.26	46	1.94	1.39–2.50
Female	2671	394	14.75	13.41–16.10	69	2.58	1.98–3.18	38	1.42	0.97–1.87	142	5.32	4.47–6.17	145	5.4	4.57–6.29
Age (years)																
60–69	2531	290	11.46	10.22–12.70	56	2.21	1.64–2.79	29	1.15	1.54–2.65	91	3.60	2.87–4.32	114	4.5	3.70–5.31
70–79	1632	158	9.68	8.25–11.12	33	2.02	1.34–2.71	8	0.49	0.15–0.83	61	3.74	2.82–4.66	56	3.43	2.82–4.66
80+	875	71	8.11	6.30–9.92	13	1.49	0.68–2.29	6	0.69	0.14–1.23	31	3.54	2.32–4.77	21	2.40	1.39–3.41
Education																
Illiterate	2028	225	11.09	9.73–12.46	32	1.55	1.04–2.12	19	0.94	0.52–1.36	88	4.34	3.43–5.23	86	4.24	3.36–5.12
≤ 5 years	1394	151	10.8	9.20–12.46	19	1.36	0.75–1.97	14	1.00	0.48–1.53	66	4.73	3.62–5.85	52	3.73	2.74–4.73
> 5 years	1616	143	8.85	7.46–10.23	51	3.16	2.30–4.01	10	0.62	0.24–1.00	29	1.79	1.15–2.44	53	3.30	2.41–4.15

^a^ *N* number of individuals included in analysis, *TTH* tension-type headaches, *MIG* migraines, *CH* chronic headache (headache ≥15 days/month), *UNC* unclassified headaches, ^b^ *Prev* prevalence of headache, ^c^CI: confidence interval

The 1-year prevalence of primary headaches, TTH, MIG, CH, and UNC in males are 5.28 % (95 % CI 4.38–6.18), 1.39 % (95 % CI 0.92–1.87), 0.21 % (95 % CI 0.03–0.40), 1.94 % (95 % CI 1.39–2.50) and 1.73 % (95 % CI 1.21–2.26). The observed 1-year prevalence for primary headaches, TTH, MIG, CH, and UNC in females is 14.75 % (95 % CI 13.41–16.10), 2.58 % (95 % CI 1.98–3.18), 1.42 % (95 % CI 0.97–1.87), 5.40 % (95 % CI 4.57–6.29)and 5.32 % (95 % CI 4.47–6.17).In a word,the 1-year prevalence of primary headache was higher in women than in men.

Among the 2531 participants between 60 and 69 years of age, the 1-year prevalence of primary headache, TTH, MIG, CH, and UNC are 11.46 % (95 % CI 10.22–12.70),2.21 % (95 % CI 1.64–2.79),1.15 % (95 % CI 1.54–2.65),4.5 % (95 % CI 3.70–5.31) and 3.6 % (95 % CI 2.87–4.32). Among the 1632 residents who were between 70 and 79 years old, the 1-year prevalence for all primary headaches,TTH, MIG, CH, and UNC are 9.68 % (95 % CI 8.25–11.12), 2.02 % (95 % CI 1.34–2.71), 0.49 % (95 % CI 0.15–0.83), 3.43 % (95 % CI 2.82–4.66), 3.74 % (95 % CI 2.82–4.66). Among the 875 participants who were 80 and older, the 1-year prevalence of primary headache, TTH, MIG, CH and UNC are 8.11 % (95 % CI 6.30–9.92), 1.49 % (95 % CI 0.68–2.29), 0.69 % (95 % CI 0.14–1.23), 2.4 % (95 % CI 1.39–3.41) and 3.54 % (95 % CI 2.32–4.77). In brief, the 1-year prevalence of primary headache was decreased with increasing age.

Among the 2028 participants whose educational level is illiterate, the 1-year prevalence for primary headache, TTH, MIG, CH and UNC are 11.09 % (95 % CI 9.73–12.46), 1.55 % (95 % CI 1.04–2.12), 0.94 % (95 % CI 0.52–1.36),4.24 % (95 % CI 3.36–5.12) and 4.34 % (95 % CI 3.43–5.23). Among the 1394 residents whose education level is less than or equal to 5 years, the 1-year prevalence for primary headache, TTH, MIG, CH and UNC are 10.80 % (95 % CI 9.20–12.46), 1.36 % (95 % CI 0.75–1.97), 1.00 % (95 % CI 0.48–1.53), 3.73 % (95 % CI 2.74–4.73) and 4.73 % (95 % CI 3.62–5.85). Among the 1616 participants whose education level is more than 5 years, the 1-year prevalence in this group for primary headache, TTH, MIG, CH and UNC are 8.85 % (95 % CI 7.46–10.23), 3.16 % (95 % CI 2.30–4.01), 0.62 % (95 % CI 0.24–1.00), 3.30 % (95 % CI 2.41–4.15) and 1.79 % (95 % CI 1.15–2.44). The 1-year prevalence of TTH and CH were decreased with higher education.

Among the 5038 participants interviewed who were 60 years of age and older, 519 reported primary headaches. The characteristics of these primary headaches are summarized in Table 2. The majority (52.02 %) of participants who experienced primary headache reported feeling distending pain and tightening. The most common headache duration in our study population was less than 4 h but greater than or equal to 30 min (27.36 %), although the greatest percentage of those with primary headache was unable to determine the duration (42.77 %). Similarly, while phonophobia and/or photophobia were the most commonly associated symptoms (31.80 %), most respondents (51.45 %) reported no associated symptoms. Regarding attack frequency, the largest group (41.62 %) experienced headache less than 1 day per month. Physical activity was not an aggravating factor for the majority (79.90 %) of participants with primary headache, and most (51.45 %) reported a normal daily life. More participants reported unilateral (48.85 %) than bilateral (34.87 %) headache symptoms. Regarding headache severity, the majority (60.3 %) of those with primary headache were given a VAS score of 1 to 3, corresponding to a mild headache. In a word, headache in the elderly was mild to moderate and not serious (Table 2).

**Table 2:** Number and percentage of participants reporting various primary headache characteristics

	N (%)
Total (5038)	519(10.30)
Headache characteristic	
Pulsating	66(12.72)
Aching	69(13.29)
Distending pain and tightening	270(52.02)
Others	114(21.97)
Attack duration	
< 30 min	64(12.33)
30 min to 4 h(≥30 min and <4 h)	142(27.36)
4–72 h (≥4 h and <72 h)	43(8.29)
≥ 72 h	48(9.25)
Undetermined	222(42.77)
Associated symptoms	
Nausea and/or vomiting	84(16.18)
Phonophobia and/or photophobia	165 (31.80)
Lacrimation/conjunctival injection	3(0.58)
Not reported	267(51.44)
Headache days	
Occasional (<1 day/month)	216(41.62)
Episodic (>1 day/month& < 15 days/month)	112 (21.58)
Chronic (≥15 days/month)	94(18.11)
Attack everyday	97(18.70)
Headache aggravated by physical activity	
Yes	104(20.10)
No	415(79.90)
Effect on daily life	
Daily life was normal	267(51.45)
Part of daily life was affected	201(38.72)
Could not do anything	51(9.83)
Number of kinds of headache	
One kind	366(70.52)
Two kinds or more	54(10.40)
Indeterminate	99(19.08)
Location of headache	
Unilateral	254(48.95)
Bilateral	181(34.87)
Indeterminate	84(16.18)
Similar attack in family history	
Yes	61(11.75)
No	369(71.10)
Indeterminate	89(17.15)
VAS score	
1–3(≥1& ≤ 3)	313(60.30)
4–6(≥4& ≤ 6)	158(30.44)
≥ 7	48(9.26)

According to univariate analysis, we found significant associations between sex, age, education, smoking, drinking, widowed/separated marital status, social activities, hypertension, diabetes mellitus, heart disease and primary headaches. A subsequent multivariate analysis found that independent risk factors for primary headaches were female gender (OR = 2.906; 95 % CI: 1.833–4.608) and engagement in social activities (OR = 2.157, 95 % CI: 1.072–4.339) and accompany with heart disease (OR =1.949; 95 % CI: 1.354–2.804) (Tables 3 and 4).

**Table 3:** Univariate analysis comparing variables significantly associated with primary headache against healthy respondents

	Healthy participants	Participants with primary headache	*P* value
	n (%)	n (%)	
	4519	519	
Sex			
Male	2241(49.6)	125(24.1)	*P* < 0.001*
Female	2278(50.4)	394(75.9)	
Age			
60–69	2243(49.6)	290(55.9)	*P* = 0.007*
≥ 70	2276(50.4)	229(44.1)	
Education (years)			
≤ 5	3040(67.3)	376(72.4)	*P* = .017*
> 5	1479(32.7)	143(27.6)	
Current smoker			
Yes	1338(29.6)	86(16.6)	*P* < 0.001*
No	3181(70.4)	433(83.4)	
Currently drinks alcohol			
Yes	1164(25.8)	62(11.9)	*P* < 0.001*
No	3355(74.2)	457(88.1)	
Widowed/separated			
Yes	791(17.5)	79(15.2)	*P* = 0.033*
No	3728(82.5)	440(84.8)	
Social activities			
Yes	793(17.5)	29(5.6)	*P* < 0.001*
No	3726(82.5)	490(94.4)	
Comorbidities			
Hypertension	1520(33.6)	209(40.3)	*P* = 0.003*
	2999(66.4)	310(59.7)	
Diabetes mellitus	337(7.5)	55(10.6)	*P* = 0.011*
	4182(92.5)	464(89.4)	
Heart disease	631(14)	140(27)	*P* < 0.001*
	3888(86)	379(73)	

*It has statistical significance

**Table 4:** Multivariate analysis of variables significantly associated with primary headache compared with healthy respondents

	B	OR (95 % CI)	*P* value
Sex – female	1.067	2.906(1.833–4.608)	<0.01*
Age	−0.157	0.854(0.607–1.202)	0.367
Education	−0.138	0.871(0.608–1.247)	0.451
Smokes cigarettes	−0.015	0.985(0.787–1.234)	0.896
Drinks alcohol	−0.173	0.841(0.490–1.443)	0.530
Widowed/separated	0.317	1.373(0.877–2.148)	0.166
Engages in social activities	0.769	2.157(1.072–4.339)	0.031*
Hypertension	0.260	1.297(0.935–1.799)	0.120
Diabetes mellitus	−0.056	0.946(0.553–1.618)	0.839
Heart disease	0.667	1.949(1.354–2.804)	<0.01*

*It has statistical significance

According to univariate analysis, we found sex, education, drinking, social activities, diabetes mellitus, and heart disease to be significantly associated with TTH, as compared to healthy subjects. A subsequent multivariate analysis found that independent risk factors for TTH were education level (OR =2.028, 95 % CI: 1.016–4.048), and accompany with heart disease (OR =3.150; 95 % CI: 1.637–6.063) (Tables 5 and 6).

**Table 5:** Univariate analysis comparing variables significantly associated with TTH against healthy respondents

	Healthy participants	Participants with TTH	*P* value
	n (%)	n (%)	
	4519	102	
Sex			
Male	2241(49.6)	33(32.4)	0.001*
Female	2278(50.4)	69(67.6)	
Age			
60–69	2243(49.6)	56(54.9)	0.293
≥ 70	2276(50.4)	46(45.1)	
Education (years)			
≤ 5	3040(67.3)	51(50)	*P* < 0.001*
> 5	1479(32.7)	51(50)	
Current smoker			
Yes	1338(29.6)	23(22.5)	0.122
No	3181(70.4)	79(77.5)	
Currently drinks alcohol			
Yes	1164(25.8)	11(10.8)	*P* < 0.001*
No	3355(74.2)	91(89.2)	
Widowed/separated			
Yes	791(17.5)	14(13.7)	0.320
No	3728(82.5)	88(86.3)	
Social activities			
Yes	793(17.5)	6(5.9)	*P* = 0.002*
No	3726(82.5)	96(94.1)	
Comorbidities			
Hypertension	1520(33.6)	43(42.2)	0.072
	2999(66.4)	59(57.8)	
Diabetes mellitus	337(7.5)	17(16.7)	0.001*
	4182(92.5)	85(83.3)	
Heart disease	631(14)	40(39.2)	*P* < 0.01*
	3888(86)	62(60.8)	

*It has statistical significance

**Table 6:** Multivariate analysis comparing variables significantly associated with TTH against healthy participants

	B	OR (95 % CI)	*P* value
Sex – female	0.305	1.356(0.604–3.043)	0.460
Education (>5 years)	0.707	2.028(1.016–4.048)	0.045*
Drinks alcohol	−1.094	0.335(0.104–1.080)	0.067
Social activities	0.638	1.892(0.447–8.007)	0.386
Diabetes mellitus	0.586	1.797(0.718–4.497)	0.210
Heart disease	1.147	3.150(1.637–6.063)	0.001*

*It has statistical significance

According to univariate analysis, we observed that sex, age, and drinking were significantly associated with MIG when compared with healthy subjects. A subsequent multivariate analysis found that independent risk factor for MIG was female gender (OR = 6.548; 95 % CI: 1.053–40.736) (Tables 7 and 8).

**Table 7:** Univariate analysis comparing variables significantly associated with MIG against healthy respondents

	Healthy participants	Participants with MIG	*P* value
	n (%)	n (%)	
	4519	43	
Sex			
Male	2241(49.6)	5(11.6)	*P* < 0.01*
Female	2278(50.4)	38(88.4)	
Age			
60–69	2243(49.6)	29(67.4)	*P* = 0.02*
≥ 70	2276(50.4)	14(32.6)	
Education (years)			
≤ 5	3040(67.3)	33(76.7)	*P* = 0.187
> 5	1479(32.7)	10(23.3)	
Current smoker			
Yes	1338(29.6)	10(23.3)	*P* = 0.364
No	3181(70.4)	33(76.6)	
Currently drinks alcohol			
Yes	1164(25.8)	5(11.6)	*P* = 0.035*
No	3355(74.2)	38(88.4)	
Widowed/separated			
Yes	791(17.5)	8(18.6)	*P* = 0.85
No	3728(82.5)	35(81.4)	
Social activities			
Yes	793(17.5)	5(11.6)	*P* = 0.309
No	3726(82.5)	38(88.4)	
Comorbidities			
Hypertension	1520(33.6)	15(34.9)	*P* = 0.863
	2999(66.4)	28(65.1)	
Diabetes mellitus	337(7.5)	5(11.6)	*P* = 0.301
	4182(92.5)	38(88.4)	
Heart disease	631(14)	8(18.6)	*P* = 0.383
	3888(86)	35(81.4)	

*It has statistical significance

**Table 8:** Multivariate analysis of variables significantly associated with MIGagainst healthy participants

	B	OR (95 % CI)	*P* value
Age	−0.780	0.458(0.143–1.470)	0.189
Sex – female	1.879	6.548(1.053–40.736)	0.044*
Drinks alcohol	0.391	1.479(0.237–9.219)	0.675

*It has statistical significance

According to univariate analysis, we found sex, age, smoking, drinking, social activities, hypertension, diabetes mellitus, and heart disease to be significantly associated with CH versus with healthy subjects. A subsequent multivariate analysis found that independent risk factors for CH were female gender (OR = 2.334; 95 % CI: 1.222–4.460) and accompany with heart disease (OR = 1.882; 95 % CI: 1.119–3.166) (Tables 9 and 10).

**Table 9:** Univariate analysis comparing variables significantly associated with CH against healthy participants

	Healthy participants	Participants with CH	*P* value
	n (%)	n (%)	
	4519	191	
Sex			
Male	2241(49.6)	46(24.1)	*P* < 0.01*
Female	2278(50.4)	145(75.9)	
Age			
60–69	2243(49.6)	114(59.7)	*P* = 0.007*
≥ 70	2276(50.4)	77(40.3)	
Education (years)			
≤ 5	3040(67.3)	138(72.3)	*P* = 0.150
> 5	1479(32.7)	53(27.7)	
Current smoker			
Yes	1338(29.6)	36(18.8)	*P* = 0.001*
No	3181(70.4)	155(81.2)	
Currently drinks alcohol			
Yes	1164(25.8)	23(12 %)	*P* < 0.01*
No	3355(74.2)	168(88 %)	
Widowed/separated			
Yes	791(17.5)	33(17.3)	*P* = 0.963
No	3728(82.5)	158(82.7)	
Social activities			
Yes	793(17.5)	14(7.3)	*P* < 0.01*
No	3726(82.5)	177(92.7)	
Comorbidities			
Hypertension	1520(33.6)	81(42.4)	*P* = 0.012*
	2999(66.4)	110(57.6)	
Diabetes mellitus	337(7.5)	25(13.1)	*P* = 0.004*
	4182(92.5)	166(86.9)	
Heart disease	631(14)	48(25.1)	*P* < 0.01*
	3888(86)	143(74.9)	

*It has statistical significance

**Table 10:** Multivariate analysis of variables significantly associated with CH against healthy participants

	B	OR (95 % CI)	*P* value
Age	−0.227	0.797(0.495–1.282)	0.797
Sex – female	0.848	2.334(1.222–4.460)	0.01*
Engages in social activities	0.237	1.268(0.537–2.992)	0.588
Smokes cigarettes	0.013	1.013(0.841–1.220)	0.893
Drinks alcohol	−0.323	0.724(0.325–1.611)	0.428
DM	0.502	1.651(0.856–3.186)	0.135
Heart disease	0.633	1.882(1.119–3.166)	0.017*
Hypertension	0.385	1.469(0.912–2.365)	0.108

*It has statistical significance

According to univariate analysis, we observed that sex, education, smoking, drinking, social activities, and heart disease were significantly associated with UNC when compared with healthy subjects. A subsequent multivariate analysis found that independent risk factors for UNC were female gender (OR =2.695; 95 % CI: 1.396–5.2) and engagement in social activities (OR = 5.183, 95 % CI: 1.258–21.349) and accompany with heart disease (OR =1.803; 95 % CI: 1.091–2.980) (Tables 11 and 12).

**Table 11:** Univariate analysis comparing variables significantly associated with UNC against healthy participants

	Healthy participants	Participants with UNC	*P* value
	n (%)	n (%)	
	4519	183	
Sex			
Male	2241(49.6)	41(22.4)	*P* < 0.01*
Female	2278(50.4)	142(77.6)	
Age			
60–69	2243(49.6)	91(49.7)	*P* = 0.981
≥ 70	2276(50.4)	92(50.3)	
Education (years)			
≤ 5	3040(67.3)	154(84.2)	*P* < 0.01*
> 5	1479(32.7)	29(15.8)	
Current smoker			
Yes	1338(29.6)	17(9.3)	*P* < 0.01*
No	3181(70.4)	166(90.7)	
Currently drinks alcohol			
Yes	1164(25.8)	23(12.6)	*P* < 0.01*
No	3355(74.2)	160(87.4)	
Widowed/separated			
Yes	791(17.5)	24(13.1)	*P* = 0.124
No	3728(82.5)	159(86.9)	
No social activities			
Yes	793(17.5)	4(2.2)	*P* < 0.001*
No	3726(82.5)	179(97.8)	
Comorbidities			
Hypertension	1520(33.6)	70(38.3)	*P* = 0.196
	2999(66.4)	113(61.7)	
Diabetes mellitus	337(7.5)	8(4.4)	*P* = 0.117
	4182(92.5)	175(95.6)	
Heart disease	631(14)	44(24)	*P* < 0.01*
	3888(86)	139(76)	

*It has statistical significance

**Table 12:** Multivariate analysis of variables significantly associated with UNC versus individuals with healthy respondents

	B	OR (95 % CI)	*P* value
Sex – female	0.991	2.695(1.396–5.2)	0.003*
Education (>5 years)	−0.175	0.840(0.508–1.387)	0.495
Smokes cigarettes	−0.193	0.824(0.394–1.725)	0.608
Drinks alcohol	−0.038	0.963(0.437–2.124)	0.926
Engages in social activities	1.645	5.183(1.258–21.349)	0.023*
Heart disease	0.589	1.803(1.091–2.980)	0.022*

*It has statistical significance

We analyzed the variables for participants with different types of headache and found that education (*P* < 0.001), smoking (*P* = 0.008), social activities (*P* = 0.044), diabetes mellitus (*P* = 0.005), and heart disease (*P* = 0.007) were significantly different among the various types of headache. Participants whose education level averaged more than 5 years were more often predisposed to TTH than to MIG (*P* = 0.003), CH (*P* < 0.001), and UNC (*P* < 0.001). In addition, participants whose education level averaged more than 5 years were more often predisposed to CH than to UNC (*P* = 0.005). The study participants who did not smoke had a greater predisposition for UNC than for TTH (*P* = 0.002), MIG (*P* = 0.011), and CH (*P* = 0.008). Participants who reported no social activities were less predisposed to UNC than to CH (*P* = 0.02) and MIG (*P* = 0.004). Study participants who had diabetes mellitus were less often predisposed to UNC than TTH (*p* < 0.001) and CH (*p* = 0.003). The participants with heart disease showed a greater predisposition for TTH than for MIG (*P* = 0.011), CH (*p* = 0.007), and UNC (*P* = 0.004) (Table 13).

**Table 13:** Comparison of variables in respondents with various subtypes of primary headache

	Participants with headache	Participants with TTH	Participants with MIG	Participants with CH	Participants with UNC	*P* value
	n (%)	n (%)	n (%)	n (%)	n (%)	
	519	102	43	191	183	
Sex						
Male	125	33(32.4)	5(11.6)	46(24.1)	41(22.4)	*P* = 0.052
Female	394	69(67.6)	38(88.4)	145(75.9)	142(77.6)	
Age						
60–69	290	56(54.9)	29(67.4)	114(59.7)	91(49.7)	*P* = 0.098
≥70	229	46(45.1)	14(32.6)	77(40.3)	92(50.3)	
Education (years)						
≤ 5	376	51(50)	33(76.7)	138(72.3)	154(84.2)	*P* < 0.001{1vs2 *P* = 0.003} {1VS3 *P* < 0.001} {1vs4 *p* < 0.001} {2vs3 *p* = 0.548} {2vs4 *p* = 0.247} {3vs4 *p* = 0.005}
> 5	143	51(50)	10(23.3)	53(27.7)	29(15.8)	
Current smoker	86	23(22.5)	10(23.3)	36(18.8)	17(9.3)	*P* = 0.008* {1vs2 *P* = 0.926} {1vs3 *P* = 0.452} {1vs4 *P* = 0.002} {2vs3 *P* = 0.511} {2vs4 *P* = 0.011} {3vs4 *P* = 0.008}
	433	79(77.5)	33(76.6)	155(81.2)	166(90.7)	
Drinks alcohol	62	11(10.8)	5(11.6)	23(12 %)	23(12.6)	*P* = 0.977
	457	91(89.2)	38(88.4)	168(88 %)	160(87.4)	
Widowed/separated	79	14(13.7)	8(18.6)	33(17.3)	24(13.1)	*P* = 0.612
	440	88(86.3)	35(81.4)	158(82.7)	159(86.9)	
Social activities	29	6(5.9)	5(11.6)	14(7.3)	4(2.2)	*P* = 0.044* {3vs4 *p* = 0.02} {2vs4 *p* = 0.004} {1vs4 *p* = 0.104} {2vs3 *p* = 0.351} {1vs3 *P* = 0.640} {1VS2 *P* = 0.233}
	490	96(94.1)	38(88.4)	177(92.7)	179(97.8)	
Comorbidities						
Hypertension	209	43(42.2)	15(34.9)	81(42.4)	70(38.3)	*P* = 0.719
	310	59(57.8)	28(65.1)	110(57.6)	113(61.7)	
Diabetes mellitus	55	17(16.7)	5(11.6)	25(13.1)	8(4.4)	*P* = 0.005* {1vs2 *P* = 0.440} {1vs3 *P* = 0.450} {1vs4 *P* < 0.001} {2vs3 *P* = 0.796} {2vs4 *P* = 0.066} {3vs4 *P* = 0.003}
	464	85(83.3)	38(88.4)	166(86.9)	175(95.6)	
Heart disease	140	40(39.2)	8(18.6)	48(25.1)	44(24)	*P* = 0.007* {1vs2 *P* = 0.011} {1vs3 *P* = 0.007} {1vs4 *P* = 0.004} {2vs3 *P* = 0.365} {2vs4 *P* = 0.446} {3vs4 *P* = 0.807}
	379	62(60.8)	35(81.4)	143(74.9)	139(76)	

*It has statistical significance

1, TTH; 2, MIG; 3, CH 4, UNC

## Discussion

Our study focused on the prevalence of primary headache in an elderly population in rural, northern China. While primary headache prevalence has been studied extensively, limited data exist for this population. According to our results, the prevalence of primary headache disorders among this population was 10.30 %. In addition, we found prevalence rates of 2.02 % for TTH, 0.85 % for MIG, 3.79 % for CH, and 3.63 % for UNC among all study participants. Other studies, which include data from younger participants, have suggested much higher rates of primary headache. Stovner et al. reported global lifetime prevalence rates of 66 % (65 % in males, 69 % in females) for primary headache, 14 % for MIG, and 46 % for TTH, as well as global 1-year prevalence rates of 47 % (37 % in males, 52 % in females) for primary headache in general, 12–15 % for MIG, and 38 % for TTH [1]. In a Malaysian population, one study observed 1-year prevalence rates of 26.5 % for TTH, 9.0 % for MIG, and 28.2 % for other headache subtypes [14]. One study conducted in China found 1-year prevalence rates of 23.8 % for all primary headaches, 9.3 % for MIG, and 10.8 % for TTH [6]. Compared with studies conducted on the general population, we observed much lower prevalence rates in an elderly population. Our findings are in agreement with multiple epidemiological studies that have demonstrated that primary headache prevalence decreases in elderly populations [5, 8, 9].

For TTH, studies have reported a 1-year prevalence of 16.2 % in Korea [15],and a lifetime prevalence rate of 38.3 % in Germany [16]. Another study estimates the prevalence of TTH in developed countries to be 66.7 % in males and over 80 % in females [17]. While Zhao et al. report that the peak incidence of TTH occurs between the ages of 30 and 39 in men and women in China [18], this form of primary headache is still the most common type among the elderly [19, 20]. Additional research regarding TTH among the elderly supports this wide variability in prevalence, as studies have found 1-year prevalence rates among individuals older than 50 years of age to be between 16 and 44 % [8, 21–24].

As stated previously, we observed a 1-year prevalence of 2.02 % for TTH among the elderly population of Ji County. In our study, 1.39 % of male participants and 2.58 % of female participants reported experiencing TTH. Furthermore, 2.21 % of 60–69 year olds, 2.02 % of 70–79 year olds, and 1.49 % of participants older than 80 experienced TTH. These 1-year prevalence rates are substantially lower than those found in previous studies. In our study, as in previously published research, a greater proportion of elderly females than males reported TTH. We observed a decrease in the prevalence of TTH with age, which is also in agreement with preexisting data.

Regional variability also exists in the reported prevalence of MIG, with ranges of 1 to 22 % in Asia, 9to 16 % in North America, and 10 to 25 % in Europe [1]. Similar to that for TTH, MIG prevalence appears to be higher in women than men. Studies have reported that 6 % of men and 17 % of women in Central America and Puerto Rico [9],as well as 9 % of men and 29 % of women in Turkey [25], experienced MIG. Peak age at onset of MIG varies between countries. In China, peak age at onset of MIG was between 10 and 19 years for males, and between 20 and 29 for females [18]. One study in Taiwan observed peak MIG prevalence rates in males between 20 and 29 years old, compared with peak rates in females between the ages of 30 and 39 years [26]. In Hong Kong, MIG prevalence peaked in 25- to 34-year-old females and 45- to 54-year-old men [27],while rates in Japan peaked during the fourth decade of life in women and the second decade in men [28]. A study on an American population found a bimodal distribution for peak age at onset of MIG, noting peak periods of 25 and 50 years in women, and slightly later peaks in men [29].

Many studies have found a decreased prevalence of MIG in the elderly, and some have noted that MIG symptoms become more atypical in this population [19, 29–32]. Figures for MIG prevalence in the elderly include 4.6 % among Italians, and 0.7 and 4.7 % among Chinese men and women, respectively [21, 33]. Other studies in China also found lower rates among older participants, ranging between 0.27 and 0.76 % [34, 35]. One study, conducted in Hong Kong, reported a 0.3 % MIG prevalence in an elderly population [36].

In our study, we observed a 1-year MIG prevalence of 0.85 %. A higher percentage of females (1.42 %) experienced MIG symptoms than males (0.21 %). Epidemiological studies have reported that females have a higher predisposition than males for both TTH and MIG [37, 38], and females tend to have more headaches in all age groups [39].

When we examined the results of our questionnaire according to age, we found that 1.15 % of 60–69 year olds, 0.49 % of 70–79 year olds, and 0.69 % of participants age 80 and older reported experiencing MIG. Prevalence rates in our research are lower than many of the previously mentioned studies, but are similar to rates found in other studies of elderly individuals in China [34, 35]. Thus, our findings agree with the previous studies that observed lower prevalence of MIG in elderly populations.

The global prevalence of CDH, a severely debilitating diagnosis, has been reported as 1–2 % [1]. Unlike TTH and MIG, a number of studies have found that the prevalence of CDH increases steadily with age, and is relatively frequent among the elderly [8, 19, 40–43].

In our study, 3.79 % of participants reported CH, which is higher than the observed prevalence for both TTH and MIG. We found a 1-year prevalence of 1.94 % for males and 5.40 % for females. We also found a 1-year prevalence of CH for study participants between 60 and 69 years old of 4.5 %, 3.43 % for 70 to 79 year olds, and 2.4 % for those age 80 and older. These results suggest that CH is more common than TTH and MIG among the elderly. The results of our study also indicated that women are more likely to have CH than men. However, unlike previous studies, we observed a decrease in the prevalence of CH with age.

We also investigated the prevalence of headaches that could not be classified based on the study participants’ responses. According to previously available data, the prevalence of UNC in older individuals is higher than rates for TTH and MIG [20]. In our research, 3.63 % of participants reported symptoms of UNC. The 1-year prevalence of UNC was greater in females (5.32 %) than males (1.73 %). An analysis of UNC according to age revealed that 3.60 % of 60 to 69 year olds, 3.74 % of 70 to 79 year olds, and 3.54 % of participants age 80 and older experienced this category of headache.

Our data showed a greater prevalence of UNC than of TTH or MIG. In our study, MIG included probable MIG, and TTH included probable TTH based on the criteria described in the ICDH-3-beta. However, a possibility still existed that some probable migraine or TTH was classified as UNC. One potential explanation for this observation is that, as a result of lower average education levels, the participants in our study may have had difficulty in describing their symptoms. Alternative explanations may be that because our study population consisted of older individuals, their symptoms may be atypical due to their more complex medical histories compared with those in younger populations [20]. Females exhibited a higher prevalence of unclassified headache than males, whereas prevalence rates for this headache were similar across different age groups.

Studies conducted on rural populations found lower prevalence rates in Tanzania (23.1 %) [44], Ethiopia (3 %) [45], and Ecuador (6.8 %) [46]. These findings may be a result of the hard, physical labor that is common in rural regions [47], as well as the normal or low body mass indices that often characterize inhabitants of these regions [48]. As previously mentioned, people in rural areas tend to have less education than those in more urban settings, leading to a decreased awareness of their headache symptoms, particularly among women [49].

To date, few data exist regarding the association between education level and primary headache prevalence. In our research, although the prevalence of primary headache decreased with increasing levels of education, the results were not statistically significant. Furthermore, we found no statistically significant difference between each subtype of headache (TTH, MIG, CH, and UNC) and education level. It is possible that participants with more education were able to recognize and control their symptoms more effectively than those with less education.

However, we observed that participants whose average education level was greater than or equal to 5 years had a greater predisposition for TTH than for MIG (*p* = 0.003), CH (*p* < 0.001), and UNC (p < 0.001). Additionally, we observed an increased predisposition for TTH among participants with an education level of five or more years compared with healthy participants. Sleep disturbances, not being married, and mental states such as stress, anxiety, depression, and fatigue have been identified as risk factors for TTH [50]. It is believed that TTH disorders are the body’s physiological expression of these mental factors. Individuals with higher education levels may face greater work-related stress and have to balance a higher number of occupational and social responsibilities than people with less education, leading to an increased risk of TTH.

Women exhibited a greater predisposition than men for primary headache, MIG, CH and UNC. Women also displayed a greater tendency than men to have TTH, but the results of our multivariate regression analysis were not significant. Owing to the relationship between TTH and work-related stress, it is possible that the men in our study experienced greater levels of work-related stress, and, as a result, the prevalence of TTH between the sexes was more similar than other forms of primary headache.

We also found that respondents who had no social activities were generally less predisposed than those with social activities to primary headache and UNC. The results for the TTH data showed the same tendency, but did not meet the criteria for significance in our multivariate regression analysis. Participants with greater social demands may experience higher stress, thus providing a possible explanation for our results.

We observed significant associations between heart disease and a number of primary headaches, underscoring the importance of cardiovascular health for neurological well-being. The participants with heart disease had greater predispositions than healthy participants for primary headache, TTH, CH and UNC.The results for MIG showed the same trend but were not statistically significant.

Previous studies have found that people who experience migraines have an increased risk of cardiovascular disease [51]. Others have suggested that heart disease may lead to headache, including migraine, as well as cluster and tension headaches [52]. Winsvold et al. found that both migraine and non-migraine headaches are risk factors for cardiovascular disease [53]. Other studies have observed an increased risk of coronary artery disease among participants with migraines, although the association is less definitive [54–57].

Additionally, those who had cluster headaches were more likely to also have cardiovascular disease, especially among smokers [58]. However, one review found that migraine is not associated with heart disease [59]. Another paper reported that although coronary artery disease appeared to be more common in males with cluster headaches, the results were not statistically significant [60].

Our research supports previous studies that have observed a relationship between heart disease and primary headache, especially TTH, CH, and UNC. The mechanism for this association is unknown, but a genetic basis may exist. One study found a high degree of overlap in genetic risk loci between migraine and coronary artery disease. Migraine without aura and coronary artery disease share common biological processes [61]. The vascular system may contribute to primary headache pathogenesis, but further research is needed to improve our understanding of these disease processes.

There were several limitations in our research. Our study was limited to a rural area of northern China, and additional large prospective studies will be needed. Some of our results require additional explanation. For example, we are unable to explain the association between heart disease and primary headache. More in-depth studies will be needed in this area. We did not include potentially relevant variables, such as physical activity, income, sleep quality, body mass index, which may influence primary headache prevalence and symptoms.

## Conclusion

In a population older than 60 years of age in rural, northern China, we found the 1-year prevalence of primary headache was 10.30 %, of TTH was 2.02 %, of MIG was 0.85 %, of CH was 3.79 %, and of UNC was 3.63 %. The prevalence of all of these was much lower than those observed in previous studies examining prevalence rates in the general population. The results of our study also indicated that women are more likely to have primary headache than men. We also found that although headache prevalence decreased with advancing age, CH was frequent among the elderly. Participants with higher education levels were more predisposed than healthy participants to TTH. The headache symptoms among our elderly study population were generally moderate, and not severe. We also found that respondents who did not participate in social activities had a decreased predisposition for primary headache in general, as well as for UNC. Lastly, we found that participants who had heart disease were more likely than healthy participants to experience primary headache, TTH, CH, and unclassified headache, although the reason for this was unclear.
